# Information Fusion for Industrial Mobile Platform Safety via Track-Before-Detect Labeled Multi-Bernoulli Filter †

**DOI:** 10.3390/s19092016

**Published:** 2019-04-29

**Authors:** Tharindu Rathnayake, Ruwan Tennakoon, Amirali Khodadadian Gostar, Alireza Bab-Hadiashar, Reza Hoseinnezhad

**Affiliations:** School of Engineering, RMIT University, Melbourne, VIC 3000, Australia; tharindu.rathnayake@rmit.edu.au (T.R.); ruwan.tennakoon@rmit.edu.au (R.T.); amirali.khodadadian@rmit.edu.au (A.K.G.); abh@rmit.edu.au (A.B.-H.)

**Keywords:** multi-target tracking, random finite sets, track-before-detect, Bayesian, labeled multi bernoulli, Kullback–Leibler divergence

## Abstract

This paper presents a novel Track-Before-Detect (TBD) Labeled Multi-Bernoulli (LMB) filter tailored for industrial mobile platform safety applications. At the core of the developed solution is two techniques for fusion of color and edge information in visual tracking. We derive an application specific separable likelihood function that captures the geometric shape of the human targets wearing safety vests. We use a novel geometric shape likelihood along with a color likelihood to devise two Bayesian updates steps which fuse shape and color related information. One approach is sequential and the other is based on weighted Kullback–Leibler average (KLA). Experimental results show that the KLA based fusion variant of the proposed algorithm outperforms both the sequential update based variant and a state-of-art method in terms of the performance metrics commonly used in computer vision literature.

## 1. Introduction

Industrial mobile platforms have been in use since the industrial revolution. There are many variants of these machines such as forklifts, electric buggies, boom and scissor lifts and construction cranes. These machines generally weigh in the order of tonnes and have a high potential to inflict severe injuries or even death. For example, counterbalanced rider lift trucks (more commonly known as forklifts) are universally used in manufacturing plants, warehouses, freight terminals and trade environments because of their ability to shift heavy loads efficiently. A typical counterbalanced forklift weighs about 3 tonnes and has a 2.5 tonne lifting capacity, totaling a staggering combined weight of up to 5.5 tonnes. The above example can be extended to all other types of mobile industrial platforms.

Safety is of paramount importance and a critical element to be considered and catered for during the operation of mobile industrial platforms. During 1997–2013, in the state of Victoria, Australia, there were approximately 2500 incidence reports involving forklifts that led to injuries, near misses and material damage [1]. To improve safety, various procedural changes and standards have been gradually introduced to isolate the forklifts from workers. For example, distinct predefined paths for forklifts and pedestrians were introduced at the cost of a productive work environment and an optimized floor plan [2]. Such attempts have been shown to lead to only slight improvements in the rate of collision accidents.

Smart solutions that provide intelligent safety warning systems can significantly reduce the rate of incidences and improve safety in industrial environments where heavy mobile platforms operate. One of the first attempts was to use Radio Frequency Identification (RFID) technology [3]. Despite being low-cost, RFID-based solutions tend to generate fairly high numbers of false alarms. For example, when someone is walking away from a forklift but is within the detectable range, an RFID system can generate a false alarm. A proposed alternative smart solution is the utilization of wireless sensor networks [4]. In such solutions, a number of reference nodes are placed throughout the industrial plant and all the workers and the mobile platforms are tagged with mobile sensor nodes. In a centralized algorithm, the distances between workers and forklifts are calculated from sensor data, and used for collision avoidance [5]. This approach is costly and not universally applicable. The batteries of the active sensor nodes used to tag the pedestrians need to be charged on a regular basis, and workers need to wear those at all times. A task that can easily be overlooked.

This paper presents a novel solution based on visual tracking via video signal processing. In practice, such a technology is not costly (owing to abundance of cheap vision systems) and can be used with any industrial mobile platform. Visual multi-target tracking methods can be broadly divided into three categories. The first includes the methods that rely only on detection of targets based on appearance models. At each time step, these models are matched with sections of the image to detect the targets, and detected targets across the time steps are connected using a labeling procedure [6,7,8,9,10]. These methods only use the information embedded in each frame of the video sequence, separately, for tracking in that frame.

The methods in second category combine filtering (or tracking) with the detection process. These methods utilize both the image data and the *temporal* information that can be acquired from the image sequence. Such methods include Multiple Hypothesis Tracker [11] and Bayesian multiple-blob tracker [12]. The final category of methods only use a filtering (or tracking) module to exploit all the image-based and temporal information. This family of tracking methods are called *track-before-detect* (TBD) methods in the multi-target tracking literature. Examples of TBD solutions devised for visual tracking include a TBD particle filter [13], TBD Multi-Bernoulli filters for visual tracking [14,15] and Hidden Markov model filtering [16].

Recently, random set filtering methods have been adopted and extensively used to solve various multi-target tracking problems. Examples of such filters include the Probability Hypothesis Density (PHD) filter [17], Cardinalized PHD (CPHD) filter [18], multi-Bernoulli filter [19,20], labeled multi-Bernoulli (LMB) filter [21] and the Vo–Vo filter [22,23]. In particular, the multi-Bernoulli filter and its labeled version, the LMB filter, have also been used to devise TBD solutions for visual multi-target tracking [14,15,24]. Many theoretical and algorithmic improvements for these algorithms are proposed in the literature. Such methods include: a GM-PHD filter with false alarm detection using an irregular window [25], a variable structure multiple model GMCPHD filter [26], multiple modal PHD filters for tracking maneuvering targets [27,28,29], joint underwater target detection and tracking using the Bernoulli filter [30] and a generalized CPHD filter with a model for spawning targets [31].

In one of our previous works [24], we proved that LMB multi-target distribution is a *conjugate prior* for a *separable* likelihood function. Conjugacy means that with the particular likelihood function, if the prior multi-target distribution is LMB, then the updated multi-target distribution is also LMB. In light of this finding, we formulate our proposed LMB TBD tracking algorithm and propose two methods to fuse the color and the edge information embedded in an image sequence in the Bayesian update step. In the application focused in this study, we have the prior knowledge that (i) the targets are humans and normally in upright walking position; and (ii) they wear a mandatory safety vest. The human-shaped contour of the targets constrains the edges and the vest color constraints the color contents of the target areas in the image.

The multi-target visual tracking algorithm presented in this paper effectively uses both color and geometric information embedded in the image sequence. In a recent work [32], we presented a novel model to exploit the geometric shape-related information using a double-ellipse structure. We proposed to use a single-target state that is comprised of the location and size parameters of two ellipses and the target velocity, and formulated a separable geometric shape likelihood function. We utilized the geometric shape likelihood and a well-established separable color likelihood to recursively propagate the prior multi-target LMB density in a Bayesian filtering framework.

In this paper, we significantly extend our initial findings reported in [32] by proposing two different approaches to fuse the color-related and shape-related information. Note that the experimental results presented in [32] are also substantially extended in this paper and a comprehensive set of visual tracking metrics are computed and compared. In the first method, a two-step sequential update is proposed, in which the predicted multi-target distribution is sequentially updated using the color likelihood followed by the newly formulated shape likelihood, assuming conditional independence of the shape and color measurements. Due to post-processing operations on the multi-Bernoulli distribution, such as pruning and merging, this assumption is not entirely accurate. Hence, in an alternative information theoretic approach for the update step of the multi-target filter, we propose to separately update the multi-target density twice, once using the geometric shape and once with the color measurements. The two posteriors are then fused in such a way that the resulting posterior density has the shortest combined distance (measured using weighted Kullback–Leibler average—KLA) from the initial densities, and thus incorporates the information contents of the updated posteriors based on their consensus.

The key contributions of this work are (i) devising an information theoretic method (based on minimizing the weighted Kullback–Leibler average) for fusion of color and shape measurements in a multi-Bernoulli visual tracking framework, and comparing it with the common fusion method based on sequential update as well as with a state-of-the-art method; and (ii) a comprehensive overall labeled multi-Bernoulli visual tracking solution that is particularly tailored for people tracking in industrial environments.

Experimental results involving visual tracking of industry workers show that our proposed methods outperform the state of the art in terms of false negatives and tracking performance in presence of occlusions. This is while our methods perform similar to the compared techniques in terms of false positives. It is important to note that, in safety critical applications, the rate of false negatives is significantly more important than false positives, as the occurrence of overlooking a human target can lead to catastrophic outcomes.

The outline of this paper is as follows. In Section 2, we briefly review the necessary mathematical tools to describe random finite sets (RFSs) and the Labeled multi-Bernoulli (LMB) filter. Section 3 describes how an LMB filter can be devised for accurate visual tracking of workers, detailing the derivation of the separable likelihood functions that capture color and shape information contents of image data separately. Then, the two-step sequential update for fusing color and shape information is presented, followed by the minimum KLA distance-based fusion of color and shape information. In Section 4, sequential Monte Carlo (SMC) implementation of the proposed visual tracking algorithms is presented. In Section 5, the performance of the proposed algorithm is compared with the state-of-the-art in challenging scenarios involving detection and tracking of multiple moving, mixing and overlapping industry workers in a video dataset created by the authors. Section 6 is dedicated to conclusive remarks.

## 2. Background

Since the development of random finite sets (RFSs) and finite set statistics (FISST) by Mahler [17,20], random set-based multi-object filters have attracted substantial interest from the multi-target tracking community. An RFS can be intuitively described as a set of random elements with random but finite cardinality. The elements correspond to a spatial point pattern on the space of interest. Intuitively, if we use the cardinality of an RFS to represent the number of targets in the environment of interest and the values of the elements of the RFS to represent the states of targets, we can successfully develop a multi-target tracking solution in the FISST framework. This section presents an overview of the notations and definitions used in derivations and formulation of the RFS-based method presented in this paper.

### 2.1. Notation

In this paper, we use lower-case letters to represent single-object states (e.g., xandx), upper-case letters to represent multi-object states (e.g., XandX), blackboard bold letters to represent the spaces (e.g., N,XandL) and bold letters (e.g., xandX) to denote labeled entities, so that they are distinguishable from the unlabeled entities.

Furthermore, we use the standard inner product notation 〈f,g〉≜∫f(x)g(x)dx and the multi-object exponential for a real valued function h(·) raised to a set *X* which is defined as $h^{X}≜\prod_{x\in X}h\left(x\right)$, where $h^{\emptyset }≜1$ and the elements of *X* may be of any type such as scalar, vector or set, provided that the function *h* takes an argument of that type. The generalized Kronecker delta function $\delta_{Y}\left(X\right)$ and a generalization of the inclusion function $1_{Y}\left(X\right)$ are defined in [33] [TABLE I (NOTATION)].

### 2.2. Labeled RFS

A labeled RFS with state space X and discrete label space L is an RFS X on X×L such that L:X×L→L is the projection L((x,ℓ))=ℓ. The finite subset **X** of X×L has distinct labels if and only if **X** and its labels L(X)={L(x):x∈X} have the same cardinality, which can be mathematically denoted as $\delta_{\left|X\right|}L\left(x\right)=▵X=1$ or |L(X)|=|X|.

The density of a labeled RFS X is a function

$$\pi :F\left(X\times L\right)\to R^{+}\cup \left\{0\right\}$$

with unit integration over the labeled multi-object state space and the set integral defined in [22]. In order to attach a unique label to each target, each state x∈X is coupled with a unique label $\left(\ell_{t},\ell_{b}\right)\in L=\left\{\alpha_{i}:i\in N\right\}$, where N denotes the set of positive integers and all the $\alpha_{i}$’s are distinct [22]. Here, $\ell_{t}$ is the time stamp at which the target is born and $\ell_{b}$ is used to distinguish targets born at the same time.

Henceforward, for notational succinctness, we will drop the “given observation parts” ($|y_{1:k}$ and $|y_{1:(k+1)}$) of the density arguments, bearing in mind that new posteriors are dependent on the past densities and therefore observations.

### 2.3. LMB Filter

In LMB filter, the LMB RFS (described below) is recursively propagated and updated using Chapman–Kolmogorov and Bayesian update equations [23].

An LMB RFS is a multi-Bernoulli RFS augmented with labels corresponding to the successful non-empty Bernoulli components. An LMB RFS X with state space X and label space L is parametrized with a finite parameter set $\{\left(r^{\left(\varsigma \right)},p^{\left(\varsigma \right)}\right)\}:\varsigma \in \Psi$, where Ψ is the index set with its components ς are assumed to be statistically independent. If the Bernoulli component $\left(r^{\left(\varsigma \right)},p^{\left(\varsigma \right)}\right)$ yields a non-empty set, then the label of the corresponding state is given by α(ς), where α:Ψ→ L is a 1-1 mapping [23]. The set of unlabeled states is a multi-Bernoulli RFS on X. The LMB density with the above-mentioned parameters is given by [23]:

(1) $$\pi \left(X\right)=\Delta \left(X\right)1_{\alpha (\Psi )}\left(L\left(X\right)\right){\left[\Phi \left(X;\cdot \right)\right]}^{\Psi },$$

where

$$\begin{array}{cc} \Phi (X;\varsigma ) & =\left\{\begin{array}{cc} 1-r^{\left(\varsigma \right)}, & \mathrm{if}\;\alpha (\varsigma )\notin L(X),\\ r^{\left(\varsigma \right)}p^{\left(\varsigma \right)}\left(x\right), & \mathrm{otherwise}, \end{array}\right. \end{array}$$

in which *x* is extracted from X by finding the member whose label matches α(ς).

Assuming that α mapping is an identity mapping, a compact representation of the above multi-target density can be written as:

(2) $$\pi \left(X\right)=▵\left(X\right)w\left(L\left(X\right)\right)p^{X},$$

where

(3) $$\begin{array}{ccc} w(L) & = & \prod_{i\in L}\left(1-r^{\left(i\right)}\right)\prod_{\ell \in L}\frac{1_{L}\left(\ell \right)\;r^{\left(\ell \right)}}{1-r^{\left(\ell \right)}}, \end{array}$$

(4) $$\begin{array}{ccc} p(x,\ell ) & = & p^{\left(\ell \right)}\left(x\right). \end{array}$$

For simplicity of the notation, we compactly denote the above LMB density by $\pi ={\left\{r^{\left(\ell \right)},p^{\left(\ell \right)}\right\}}_{\ell \in L}$.

#### 2.3.1. LMB Prediction

Suppose, at time k−1, that the labeled multi-target state is given by $X_{k-1}$. Each state $x_{k-1}\in X_{k-1}$ may either survive to the next time step *k* with a survival probability $p_{S,k}\left(x_{k-1}\right)$ and take on a new state $x_{k}$ with probability density $f_{k|k-1}\left(x_{k}|x_{k-1}\right)$, or it may vanish with a probability $1-p_{s,k}\left(x_{k-1}\right)$. We implement this using an LMB RFS $S_{k|k-1}\left(x_{k-1}\right)$ with survival probability $r=p_{S,k}\left(x_{k-1}\right)$ and probability distribution $p\left(\cdot \right)=f_{k|k-1}\left(\cdot |x_{k-1}\right)$. Thus, according to [17,20], the labeled multi-target state $X_{k}$ at time *k* can be written as

(5) $$X_{k}=\left[\cup_{x_{k-1}\in X_{k-1}}S_{k|k-1}\left(x_{k-1}\right)\right]\cup \Gamma_{k},$$

where $\Gamma_{k}$ is the labeled multi-Bernoulli RFS of spontaneous births, which is used to detect the newly born targets at each time step *k*:

$$L_{0:k-1}\cap L_{k}=\emptyset .$$

Consider an LMB multi-object density defined in state space X and label space $L_{0:k-1}$, and parametrized by $\pi ={\left\{r^{\left(\ell \right)},p^{\left(\ell \right)}\right\}}_{\ell \in L_{0:k-1}}$. With the LMB birth model with parameters $\pi_{B}={\left\{r_{B}^{\left(\ell \right)},p_{B}^{\left(\ell \right)}\right\}}_{\ell \in L_{k}}$, described in Reuter et al. [21], the predicted multi-object density (5) is also LMB with parameters:

(6) $$\begin{array}{ccc} r_{+,S}^{\left(\ell \right)} & = & \eta_{S}^{\left(\ell \right)}\;r^{\left(\ell \right)}, \end{array}$$

(7) $$\begin{array}{ccc} p_{+,S}^{\left(\ell \right)}\left(x\right) & = & \langle p_{S}\left(\cdot ,\ell \right)f\left(x|\cdot ,\ell \right),p^{\left(\ell \right)}\left(\cdot \right)\rangle /\eta_{S}^{\left(\ell \right)}. \end{array}$$

Here, $p_{S}\left(x,\ell \right)$ is the state-dependent *probability of survival* for an existing Bernoulli component with label *ℓ*, $f(x_{k+1}|x_{k},\ell )$ is the single-object transition density, and $\eta_{S}\left(\ell \right)=\langle p_{S}\left(\cdot ,\ell \right),p^{\left(\ell \right)}\left(\cdot \right)\rangle$.

#### 2.3.2. TBD-LMB Update

It was proved in [prop. 1] [24] that, if a measurement likelihood function can be formulated in the following *separable* form (for an image observation *y* given the labeled multi-target state X):

(8) $$g\left(y|X\right)=f\left(y\right)\prod_{(x,\ell )\in X}g_{y}\left(x,\ell \right),$$

and if the prior multi-target density is LMB, then applying Bayes rule (i.e., Bayes update) leads to a posterior that is LMB as well, i.e., LMB density is a conjugate prior with the above separable likelihood function. Indeed, if the prior is parametrized by $\pi ={\left\{r^{\left(\ell \right)},p^{\left(\ell \right)}\right\}}_{\ell \in L}$, the density given by Equations (2)–(4) turns into an LMB posterior given by:

(9) $$\pi \left(X|y\right)\propto \Delta \left(X\right)w_{y}\left(L\left(X\right)\right){\left[p(\cdot |y)\right]}^{X},$$

where

(10) $$\begin{array}{ccc} w_{y}\left(L\right) & = & {\left[\eta_{y}\right]}^{L}\;w\left(L\right), \end{array}$$

(11) $$\begin{array}{ccc} p(x,\ell |y) & = & \frac{p^{\left(\ell \right)}\left(x\right)g_{y}\left(x,\ell \right)}{\eta_{y}\left(\ell \right)}, \end{array}$$

(12) $$\begin{array}{ccc} \eta_{y}\left(\ell \right) & = & \langle p^{\left(\ell \right)}\left(\cdot \right),g_{y}\left(\cdot ,\ell \right)\rangle . \end{array}$$

The above posterior can be parametrized as $\pi_{\mathrm{updated}}={\left\{r_{\mathrm{updated}}^{\left(\ell \right)},p_{\mathrm{updated}}^{\left(\ell \right)}\right\}}_{\ell \in L}$ where

(13) $$\begin{array}{ccc} r_{\mathrm{updated}}^{\left(\ell \right)} & = & \frac{r^{\left(\ell \right)}\;\langle p^{\left(\ell \right)}\left(\cdot \right),g_{y}\left(\cdot ,\ell \right)\rangle }{1-r^{\left(\ell \right)}+r^{\left(\ell \right)}\;\langle p^{\left(\ell \right)}\left(\cdot \right),g_{y}\left(\cdot ,\ell \right)\rangle }, \end{array}$$

(14) $$\begin{array}{ccc} p_{\mathrm{updated}}^{\left(\ell \right)}\left(x\right) & = & \frac{p^{\left(\ell \right)}\left(x\right)\;g_{y}\left(x,\ell \right)}{\langle p^{\left(\ell \right)}\left(\cdot \right),g_{y}\left(\cdot ,\ell \right)\rangle }. \end{array}$$

## 3. TBD-LMB Filter for Human Tracking

This section outlines the target appearance model used to exploit the geometric shape information embedded in the image measurements. We derive the geometric shape and color likelihood functions based on the new appearance model, in order to be utilized in the LMB update step of our multi-target tracking solutions.

### 3.1. Appearance Model

**Figure 1:**
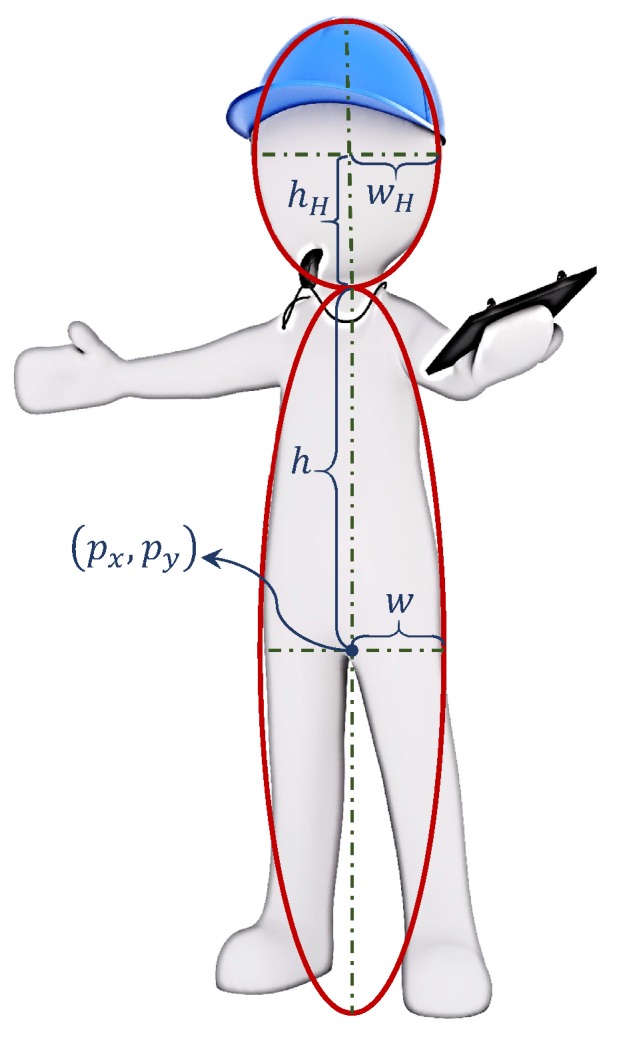
The model used for single human targets in the visual tracking for industrial mobile platform safety.

In this work, we are interested in tracking human targets who are wearing a “high visible” safety vest in an industrial environment. In such environments, it is reasonable to assume that the workers almost always move in a stand-up position. In order to capture the geometry of a human’s straight body, we model a single human target as a combination of two adjacent ellipses that share the same vertical axis, as shown in Figure 1. The target state space X is eight-dimensional, and each unlabeled single-target state is denoted by $x={\left[\begin{array}{cccccccc} p_{x} & p_{y} & \dot{p_{x}} & \dot{p_{y}} & w & h & w_{H} & h_{H} \end{array}\right]}^{⊤}$.

Generally, in other target tracking approaches such as [24,34], the target state is modeled as a rectangular blob in 2D cases (in 3D, it is generally a cube). Our formulation of the double-ellipse structure for single target state permits the formulation of separable likelihood functions for our image measurements as described in the next section. It is important to note that, as long as the likelihood function is of the separable form (8), there is no need to formulate the term f(y), as it does not appear in the update Equations (13) and (14). We only need to formulate the single-target-dependent term $g_{y}\left(\cdot \right)$.

### 3.2. Measurement Likelihood—Shape

The intuition behind our formulation of the shape likelihood is that, if the target state is accurately hypothesized (during the prediction step), there should be a substantial number of edges (in the corresponding edge-detected image measurement) around the boundary of the hypothesized target state. These edges can be approximated using the above described double-ellipse structure. Our geometric shape likelihood is built upon this intuition and it is described in the following.

The well known Canny edge detector is used on each image measurement *y*. After the prediction step at each time instance, a set of predicted targets are produced. Each target state is eight-dimensional as mentioned before and, for each hypothesized predicted target, we compute the shortest distance from every edge pixel to the particular hypothesized double-ellipse structure. As per our intuition, for a valid hypothesis (hypothesized target is close to the actual target), we expect some of the edge pixels (*inlier* pixels) to be very close to the boundary of the double-ellipse structure. These pixels can be separated from the rest (*outlier* pixels) using the Modified Selective Statistical Estimator (MSSE) [35] algorithm as described below.

Consider the *i*-th hypothesized target with unlabeled state $x_{i}$. Let us denote the shortest distance from the *j*-th edge pixel in the image to the double-ellipse boundary that represents $x_{i}$, by $d_{ij}$. Assuming that $x_{i}$ is a valid hypothesis corresponding to the correct state of an existing target, we expect that, for some edge pixels (the inliers), the distances $d_{ij}$ are small and for the rest of edge pixels (the outliers) they are large. MSSE is an effective algorithm that addresses the questions of “how small” and “how large” the distances need to be for a pixel to be labeled an inlier or outlier.

In MSSE, all the distances for the same hypothesis are first sorted in ascending order. Let us denote the sorted distance from edge pixel *j* to the boundary of hypothesis *i* by $d_{i}^{\left(j\right)}$. Naturally, the smaller distances are expected to correspond to inliers and the larger ones to outliers. Assuming that the first κ sorted distances correspond to inliers, the standard deviation of those pixels from the target boundary is given by:

(15) $$\sigma_{\kappa }^{2}=\frac{\sum_{j=1}^{\kappa }d_{i}^{\left(j\right)}}{\kappa }.$$

The next distance, which is the smallest outlier distance, is expected to be larger than $T\sigma_{\kappa }$ where *T* is a user-defined parameter in the order of a single figure (we chose T=1.90 in our experiments). Thus, in the algorithm, the above standard deviations are iteratively computed for increasing values of κ, and in each iteration it checks whether

$$d_{i}^{\left(\kappa +1\right)}>T\sigma_{\kappa }$$

is correct. If so, the algorithm stops and outputs the standard deviation of inlier distances denoted by $\sigma_{y}^{2}\left(x_{i}\right)$.

When all the standard deviations of inlier distances for all hypotheses are small, the likelihood of image measurement *y* for the given multi-target state $X={\left\{\left(x_{i},\ell_{i}\right)\right\}}_{i=1}^{n}$ should be large. We can use exponential functions to denote this mathematically as follows:

(16) $$g_{\mathrm{sh}}\left(y|{\left\{\left(x_{i},\ell_{i}\right)\right\}}_{i=1}^{n}\right)\propto \prod_{i=1}^{n}\exp\left[-\beta \;\sigma_{y}^{2}\left(x_{i}\right)\right],$$

where β is a user-defined application dependent constant. It is important to note that the proportionality factor is independent of the target states and thus the above formulated shape likelihood function follows the separable form of interest presented in Equation (8) with $g_{y_{\mathrm{sh}}}\left(x_{i},\ell \right)=\exp\left[-\beta \;\sigma_{y}^{2}\left(x_{i}\right)\right]$.

### 3.3. Measurement Likelihood—Color

Kernel density estimation over a set of histograms is one of the well known techniques to formulate color likelihoods. Following [15,36,37,38], we use kernel density estimation over a set of $n_{T}$ training HSV histograms denoted by ${\left\{h_{j}^{*}\right\}}_{j=1}^{n_{T}}$. In our experiments, $n_{T}=500$ training histograms proved to be sufficiently comprehensive. It was shown in [15] that this approach leads to the desired separable likelihood of the form (8). Indeed, the multi-target likelihood function for color contents of the image measurement, for a hypothesized multi-target state $X={\left\{\left(x_{i},\ell_{i}\right)\right\}}_{i=1}^{n}$ is given by:

(17) $$g_{\mathrm{col}}\left(y|{\left\{\left(x_{i},\ell_{i}\right)\right\}}_{i=1}^{n}\right)\propto \prod_{i=1}^{n}\frac{\epsilon }{n_{T}\times b^{n_{b}}}\sum_{j=1}^{n_{T}}\overset{˚}{k}\left(\frac{d(h_{i},h_{j}^{*})}{b}\right),$$

where ϵ is the normalization factor, $h_{i}$ is the HSV histogram of color contents of the image within the area of the hypothesized target $x_{i}$, $\overset{˚}{k}$ is the kernel function, *b* is the kernel bandwidth, $n_{b}$ is the number of bins in each histogram and $d(h_{i},h_{j}^{*})$ is the Bhattacharyya distance between the histograms [36,39,40].

Again, we note that the proportionality factor is independent of the target states and thus the above formulated color likelihood function follows the separable form of interest presented in Equation (8) with $g_{y_{\mathrm{col}}}\left(x_{i},\ell \right)=\frac{\epsilon }{n_{T}\times b^{n_{b}}}\sum_{j=1}^{n_{T}}\overset{˚}{k}\left(\frac{d(h_{i},h_{j}^{*})}{b}\right)$. Gaussian kernels are used in our experiments. Since most of the prominent color features of an image measurement are present in the luminous high visible vest, we only use the color histograms of the contents of the upper half of the lower ellipse associated with each target state.

### 3.4. Sequential Update

Instead of using a combined color-shape likelihood function in a single update step, we compute the LMB update in two steps. This way, we not only exploit both the color and shape information but also reduce the required computation as explained in the following. We note that, according to Vo et al. [41], if the measurement contents are conditionally independent, such a two-step update is theoretically equivalent to a single update step with a combined likelihood function.

The two step LMB update is as follows: we compute the color likelihood for each target using Equation (17) and the weights of particles of each hypothesized target are updated accordingly. The process of generation, prediction and update of particles is detailed as part of SMC implementation explained in Section 4. Then, in a particle pruning step, we discard the particles with weights less than a small threshold that is adaptively determined as one and half times the smallest particle weight. The remaining particles are then normalized and used in the second update step using edge likelihood given in (16).

Note that computing the edge likelihood is computationally expensive. This is because, for each target hypothesis (particle), the distances from all the numerous edge pixels to the hypothesized double-ellipse outline of the target need to be computed, then sorted, and then processed with the MSSE algorithm, and these present a much higher level of computation compared to direct calculation of color histograms and Bhattacharyya distances in the color likelihood formula. Inclusion of the pruning operation between the two update steps heavily reduces the number of particles for which the edge likelihood needs to be computed, hence substantially reducing the required computation. Indeed, our experiments showed that direct calculation of the combined likelihood is intractable in real-time applications.

### 3.5. Weighted Kla Based LMB Fusion

We propose to use Kullback–Leibler divergence which has been used in various signal processing and computer vision applications, to fuse the color related and geometric shape related information. Instances where Kullback–Leibler divergence is used in the literature include a partitioned update of a Kalman filter [42] and anomaly detection [43].

Both the double-ellipse appearance model and the formulation of the two likelihood functions are approximates. Hence, conditional independence of color and edge measurements can be inaccurate. Furthermore, in the sequential update approach, the particle pruning step contributes to the estimation error. An alternative information theoretic approach is to separately update the predicted LMB density, once using the shape and once using the color measurements, then combine the two posteriors into a fused distribution that has minimum divergence from them (and therefore encapsulates the best of information content, in terms of shape and color relevance of the hypothesized targets).

Let us denote the two LMB posteriors by

$$\pi_{\mathrm{sh}}={\left\{\left(r_{\mathrm{sh}}^{\left(\ell \right)},p_{\mathrm{sh}}^{\left(\ell \right)}\left(\cdot \right)\right)\right\}}_{\ell \in L}$$

and

$$\pi_{\mathrm{col}}={\left\{\left(r_{\mathrm{col}}^{\left(\ell \right)},p_{\mathrm{col}}^{\left(\ell \right)}\left(\cdot \right)\right)\right\}}_{\ell \in L},$$

respectively. The fused LMB density, denoted by

$$\pi ={\left\{\left(r^{\left(\ell \right)},p^{\left(\ell \right)}\left(\cdot \right)\right)\right\}}_{\ell \in L},$$

should have the smallest divergence from the above two posteriors, which is defined as the following weighted average of its Kullback–Leibler divergence from the two posteriors:

(18) $$\begin{array}{c} D_{\mathrm{KLA}}\left(\pi |\right|\pi_{\mathrm{sh}},\pi_{\mathrm{col}})≜\omega \;\;\;D_{\mathrm{KL}}\left(\pi |\right|\pi_{\mathrm{sh}})+\;\left(1-\omega \right)\;D_{\mathrm{KL}}\left(\pi |\right|\pi_{\mathrm{col}}), \end{array}$$

where ω∈[0,1] is the weight of emphasis on shape versus color information, and Kullback–Leibler divergence between two labeled RFS densities is given by:

(19) $$\begin{array}{c} D_{\mathrm{KL}}\left(\pi |\right|\pi^{\prime })≜\int \pi \left(X\right)\log\frac{\pi (X)}{\pi^{\prime }\left(X\right)}\delta X. \end{array}$$

Based on the derivations presented in (Equations (51) and (52)) [44], it is straightforward to show that the LMB density which minimizes the KLA distance (18) is parametrized as follows:

(20) $$\begin{array}{ccc} r^{\left(\ell \right)} & = & \frac{\int {\left[p_{\mathrm{sh}}^{\left(\ell \right)}\left(x\right)\right]}^{\omega }{\left[p_{\mathrm{col}}^{\left(\ell \right)}\left(x\right)\right]}^{1-\omega }dx}{\begin{array}{c} {\left[\frac{1-r_{\mathrm{sh}}^{\left(\ell \right)}}{r_{\mathrm{sh}}^{\left(\ell \right)}}\right]}^{\omega }{\left[\frac{1-r_{\mathrm{col}}^{\left(\ell \right)}}{r_{\mathrm{col}}^{\left(\ell \right)}}\right]}^{1-\omega }+\int {\left[p_{\mathrm{sh}}^{\left(\ell \right)}\left(x\right)\right]}^{\omega }{\left[p_{\mathrm{col}}^{\left(\ell \right)}\left(x\right)\right]}^{1-\omega }dx \end{array}},\\ p^{\left(\ell \right)}\left(x\right) & = & \frac{{\left[p_{\mathrm{sh}}^{\left(\ell \right)}\left(x\right)\right]}^{\omega }{\left[p_{\mathrm{col}}^{\left(\ell \right)}\left(x\right)\right]}^{1-\omega }}{\int {\left[p_{\mathrm{sh}}^{\left(\ell \right)}\left(x\right)\right]}^{\omega }{\left[p_{\mathrm{col}}^{\left(\ell \right)}\left(x\right)\right]}^{1-\omega }dx}. \end{array}$$

**Figure 2:**
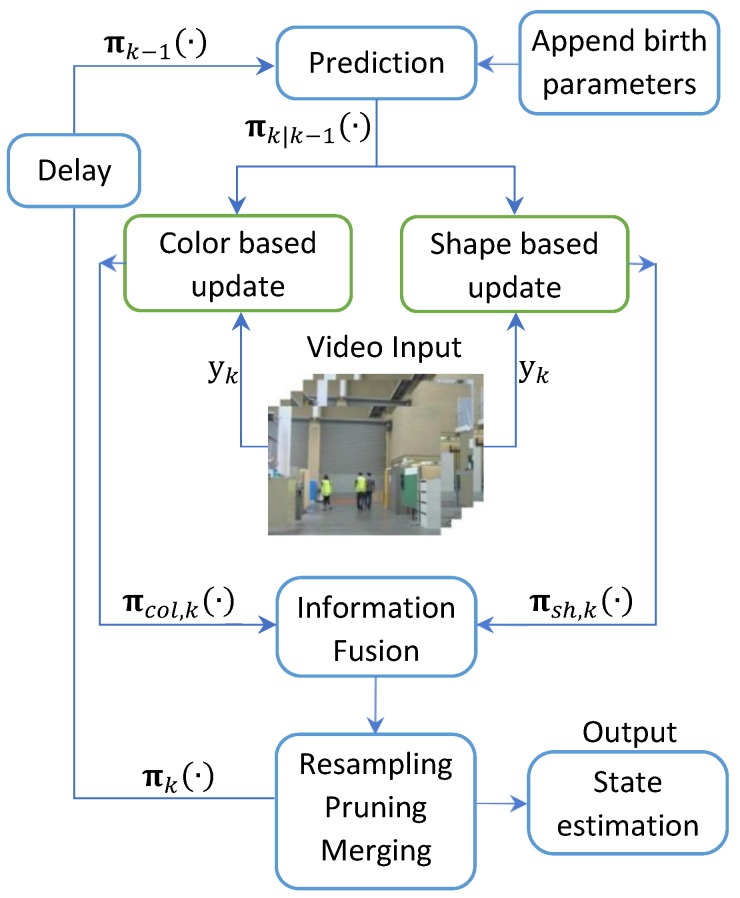
Overall diagram of the proposed algorithm.

For the sake of clarity, the overall structure of the proposed algorithm is depicted in Figure 2.

## 4. Sequential Monte Carlo (SMC) Implementation

### 4.1. SMC Prediction

Assume that, at time step *k*, the multi-target prior is parametrized as $\pi_{k-1}={\left\{r^{\left(\ell \right)},p^{\left(\ell \right)}\right\}}_{\ell \in L_{0:k-1}}$, and the density of *ℓ*-th hypothesized target, $p^{\left(\ell \right)}$, is approximated by weighted particles, i.e., $p^{\left(\ell \right)}\left(x\right)≊\sum_{j}w^{\left(\ell ,j\right)}\delta_{x^{\left(\ell ,j\right)}}\left(x\right)$. In the SMC prediction step, each particle evolves according the state transition model. In this work, we use a nearly constant velocity model for the movement of the human targets from one time step to another.

With the aforementioned particle approximation, the predicted LMB is given by [21]:

(21) $$\pi_{+}={\left\{r_{+,S}^{\left(\ell \right)},p_{+,S}^{\left(\ell \right)}\right\}}_{\ell \in L_{0:k-1}}\cup {\left\{r_{B}^{\left(\ell \right)},p_{B}^{\left(\ell \right)}\right\}}_{\ell \in L_{k}},$$

where

(22) $$\begin{array}{cc} r_{+,S}^{\left(\ell \right)} & =r^{\left(\ell \right)}\sum_{j}w^{\left(\ell ,j\right)}p_{S,k}\left(x^{\left(\ell ,j\right)}\right), \end{array}$$

(23) $$\begin{array}{cc} p_{+,S}^{\left(\ell \right)} & =\sum_{j}{\bar{w}}_{P,+}^{\left(\ell ,j\right)}\delta_{x_{+}^{\left(\ell ,j\right)}}\left(x\right), \end{array}$$

(24) $$\begin{array}{cc} r_{B}^{\left(\ell \right)} & =\mathrm{parameter}\;\mathrm{given}\;\mathrm{by}\;\mathrm{the}\;\mathrm{birth}\;\mathrm{model}, \end{array}$$

(25) $$\begin{array}{cc} p_{B}^{\left(\ell \right)} & =\sum_{j}{\bar{w}}_{B}^{\left(\ell ,j\right)}\delta_{x_{B}^{\left(\ell ,j\right)}}\left(x\right), \end{array}$$

and the evolved particles and their weights are given by [21]:

(26) $$\begin{array}{cc} x_{+}^{\left(\ell ,j\right)} & ∼q_{+}^{\left(\ell \right)}\left(\cdot |x_{B}^{\left(\ell ,j\right)},y\right), \end{array}$$

(27) $$\begin{array}{cc} w_{P,+}^{\left(\ell ,j\right)} & =\frac{w^{\left(\ell ,j\right)}f\left(x_{+}^{\left(\ell ,j\right)}|x^{\left(\ell ,j\right)}\right)p_{S,k}\left(x^{\left(\ell ,j\right)}\right)}{q^{\left(\ell \right)}\left(x_{+}^{\left(\ell ,j\right)}|x^{\left(\ell ,j\right)},y\right)}, \end{array}$$

(28) $$\begin{array}{cc} {\bar{w}}_{P,+}^{\left(\ell ,j\right)} & =w_{P,+}^{\left(\ell ,j\right)}\setminus \sum_{j}w_{P,+}^{\left(\ell ,j\right)}, \end{array}$$

(29) $$x_{B}^{\left(\ell ,j\right)}∼b_{+}^{\left(\ell \right)}\left(\cdot |y\right),$$

(30) $$w_{B}^{\left(\ell ,j\right)}=\frac{p_{B}\left(x_{B}^{\left(\ell ,j\right)}\right)}{b_{+}^{\left(\ell \right)}\left(x_{B}^{\left(\ell ,j\right)}|y\right)},$$

(31) $${\bar{w}}_{B}^{\left(\ell ,j\right)}=w_{B}^{\left(\ell ,j\right)}\setminus \sum_{j}w_{B}^{\left(\ell ,j\right)}$$

and $q_{+}^{\left(\ell \right)}\left(\cdot \right)$ and $b_{+}^{\left(\ell \right)}\left(\cdot \right)$ denote given proposal and birth densities.

### 4.2. SMC Update

Suppose that a predicted labeled multi-Bernoulli multi-object density $\pi_{+}={\left\{(r_{+}^{\left(\ell \right)},p_{+}^{\left(\ell \right)})\right\}}_{\ell \in L_{+}}$ is given with its density components $p_{+}^{\left(\ell \right)}$, being represented by a set of weighted particles,

(32) $$p_{+}^{\left(i\right)}≊\sum_{j}w_{+}^{\left(\ell ,j\right)}\delta_{x_{+}^{\left(\ell ,j\right)}}\left(x\right),$$

remembering that $L_{+}=L_{0:k}=L_{0:k-1}\cup L_{k}.$

As it was mentioned before, with the separable likelihood of the form (8), the updated labeled multi-Bernoulli multi-object parameters $\pi \left(\cdot |y\right)={\left\{(r^{\left(\ell \right)},p^{\left(\ell \right)})\right\}}_{\ell \in L_{+}}$ are given by Equations (13) and (14). With particle approximation, those turn into the following equations:

(33) $$\begin{array}{cc} r^{\left(\ell \right)} & =\frac{r_{+}^{\left(\ell \right)}\varrho_{+}^{\left(\ell \right)}}{1-r_{+}^{\left(\ell \right)}+r_{+}^{\left(\ell \right)}\varrho_{+}^{\left(\ell \right)}}, \end{array}$$

(34) $$p^{\left(\ell \right)}=\frac{1}{\varrho_{+}^{\left(\ell \right)}}\sum_{j}w_{+}^{\left(\ell ,j\right)}g_{y}\left(x_{+}^{\left(\ell ,j\right)}\right)\delta_{x_{+}^{\left(\ell ,j\right)}}\left(x\right),$$

where

(35) $$\varrho_{+}^{\left(\ell \right)}=\sum_{j}w_{+}^{\left(\ell ,j\right)}g_{y}\left(x_{+}^{\left(\ell ,j\right)}\right).$$

A simple interpretation of Equations (34) and (35) is that the updated densities retain their particles, but the weights are updated each in proportion to the $g_{y}\left(\cdot \right)$ value at the particle state, then they are renormalized to sum to 1.

In the sequential update approach, the particle weights are first updated using the color information, i.e., they are rescaled in proportion to $g_{y_{\mathrm{col}}}\left(\cdot \right)$ values. This causes the weights of numerous particles that correspond to wrong target hypotheses to reduce. Such particles are then removed in a pruning step, and the weights of the remaining particles are updated by rescaling in proportion to $g_{y_{\mathrm{sh}}}\left(\cdot \right)$ values.

In the weighted KLA based approach, the probabilities of existence and particle weights are once updated using $g_{y_{\mathrm{col}}}\left(\cdot \right)$, and once using $g_{y_{\mathrm{sh}}}\left(\cdot \right)$. This way, each probability of existence $r_{+}^{\left(\ell \right)}$ turns into two updated values denoted by $r_{\mathrm{col}}^{\left(\ell \right)}$ and $r_{\mathrm{sh}}^{\left(\ell \right)}$, and each particle weight $w_{+}^{\left(\ell ,j\right)}$ turned into two updated weights denoted by $w_{\mathrm{col}}^{\left(\ell ,j\right)}$ and $w_{\mathrm{sh}}^{\left(\ell ,j\right)}$. Based on the fundamental property of delta function, with particles, the integrals in KLA based fusion equations (20) turn into sums, and the fused probability of existence and particle weights are given by:

(36) $$\begin{array}{ccc} r^{\left(\ell \right)} & = & \frac{\sum_{j}{\left[w_{\mathrm{sh}}^{\left(\ell ,j\right)}\right]}^{\omega }{\left[w_{\mathrm{col}}^{\left(\ell ,j\right)}\right]}^{1-\omega }}{\begin{array}{c} {\left[\frac{1-r_{\mathrm{sh}}^{\left(\ell \right)}}{r_{\mathrm{sh}}^{\left(\ell \right)}}\right]}^{\omega }{\left[\frac{1-r_{\mathrm{col}}^{\left(\ell \right)}}{r_{\mathrm{col}}^{\left(\ell \right)}}\right]}^{1-\omega }+\sum_{j}{\left[w_{\mathrm{sh}}^{\left(\ell ,j\right)}\right]}^{\omega }{\left[w_{\mathrm{col}}^{\left(\ell ,j\right)}\right]}^{1-\omega } \end{array}},\\ w^{\left(\ell ,j\right)} & = & \frac{{\left[w_{\mathrm{sh}}^{\left(\ell ,j\right)}\right]}^{\omega }{\left[w_{\mathrm{col}}^{\left(\ell ,j\right)}\right]}^{1-\omega }}{\sum_{j^{\prime }}{\left[w_{\mathrm{sh}}^{\left(\ell ,j^{\prime }\right)}\right]}^{\omega }{\left[w_{\mathrm{col}}^{\left(\ell ,j^{\prime }\right)}\right]}^{1-\omega }}. \end{array}$$

### 4.3. Computational Tractability

For the sake of computational tractability, we use a number of intuitive post-processing operations such as truncating the labeled Bernoulli components with low probabilities of existence, resampling, pruning and merging. The layout of such operations does not depend on the update method (be it sequential or KLA based).

#### 4.3.1. Resampling

The particles of each Bernoulli component in the LMB posterior target distribution are resampled after the update step. The number of particles for each labeled Bernoulli component after resampling is proportional to its existence probability.

#### 4.3.2. Pruning

In the pruning step, each labeled Bernoulli component with a probability of existence less than a very small (user defined) threshold, $r_{\mathrm{th}}$ is discarded. These labeled Bernoulli components have almost zero probability to represent a true target state. In our experiments, $r_{\mathrm{th}}=0.001$. Note that this is pruning of entire Bernoulli components, and is different from pruning of low-weight particles in each Bernoulli component that is suggested as part of the sequential update approach. Without this pruning operation, due to the birth process, the number of labeled Bernoulli components would constantly increase in each filtering iteration.

#### 4.3.3. Merging

If two labeled Bernoulli components are found to be overlapped more than a user defined threshold, we merge the corresponding Bernoulli components. This is because, if they are very close to each other, there is a high probability that both the components represent the same target. In our experiments, we calculate the overlapping ratio as the ratio of the area of intersection between the two double-ellipse shapes, to the area of the smaller double-ellipse shape. If the calculated overlap ratio is greater than a user-defined threshold, we merge those two Bernoulli components. The existence probability of the resultant Bernoulli component is the sum of the existence probabilities of the two merged components, capped at 0.999. All the particles of the two merged labeled Bernoulli components are present in the resultant labeled Bernoulli component. The particle weights are scaled according to the respective probabilities of existence, then normalized. If the resultant labeled Bernoulli component has more particles than $L_{\max}$, we select the first $L_{\max}$ number of particles with the highest weights. The label of the resultant labeled Bernoulli component is chosen to be the label of the labeled Bernoulli component with the lower time stamp (the component which was generated first out of the two).

When selecting a merging threshold, one should note that a high value will result in false positives (for example, one target may be represented by multiple labeled components and since the threshold is large, these components will not merge). Similarly, a low value will result in false negatives (for example, when two targets are moving very closely to each other, a low merging threshold will merge the labeled Bernoulli component representing one target with the labeled Bernoulli component of the close by target). The application focused on in this paper is a safety critical application, and false negatives can lead to catastrophic consequences and must be avoided. Hence, the merging threshold cannot be small. In our experiments, we examined a number of choices and found a threshold of 60% to be a suitable value, returning almost no false negatives with a reasonably low rate of false positives.

### 4.4. Inference

The labeled multi-Bernoulli filter introduced in this paper is a stochastic filter in which instead of the actual set of targets, its distribution is sequentially computed. The process of obtaining estimates for the number of targets and their states from the multi-object distribution (that is sequentially predicted and updated within the filtering process) is called *inference*. We follow the common inference method used in [14,15,24,41,45]. We select the labeled Bernoulli components whose probabilities of existence are larger than a user-defined threshold. Each selected component represents a hypothesized target. The state estimate is given by the mean of its density which can be directly computed as the weighted sum of the updated particles. Indeed, the labeled set estimate is given by:

(37) $$\hat{X}=\left\{\left({\bar{x}}_{\ell },\ell \right):r^{\left(\ell \right)}>\varepsilon \right\},$$

where ε is the threshold and ${\bar{x}}_{\ell }=\sum_{j}w^{\left(\ell ,j\right)}x_{+}^{\left(\ell ,j\right)}$ in which $w^{\left(\ell ,j\right)}$ is the updated weight of the *j*-th particle of the *ℓ*-th component after resampling, merging and pruning steps.

When selecting the threshold ε, it should be noted that a high value will prune the false tracks while delaying the inclusion of new tracks, whereas a low value will include new tracks immediately (but may include some false tracks as well). As it was mentioned before, in safety critical applications, false positives are more tolerable than false negatives. Therefore, we chose a relatively low threshold of ε=0.60 in our experiments.

The other popular state estimation procedure is as follows. In the first step, the number of targets are estimated from the posterior cardinality distribution by calculating its mode (this can be achieved by calculating the mean of the cardinality distribution as well, though mode is generally preferred for its stability [19]). Then, the target tracks are ordered according to their probabilities of existence and the corresponding number of tracks with the highest probability of existence are chosen. Finally, individual modes (or means) of corresponding posterior densities are calculated to estimate the target states. Although this is a theoretically sound procedure, in a safety critical application, we do not need to omit tracks with a low probability of existence. The method used in this paper is more conservative and suited for the application at hand.

## 5. Experimental Results

We implemented our tracking algorithm in MATLAB Version R2016b, using the target state models described in Section 3.1. The targets have variable major and minor axis lengths to represent their movements towards and away from the camera. Furthermore, upper and lower bounds are defined for the axes’ lengths as well. The upper bound ensures that multiple targets are not represented by a single double-ellipse structure, and the lower bound is to make sure that the double-ellipse structure is large enough to represent a single target.

The targets are set to have a constant survival probability of $p_{S,k}\left(\cdot \right)=0.99$. We use a nearly constant velocity model for evolution of target state. The rationale behind this selection is that, in an indoor industrial environment, workers can only walk through designated paths and therefore, their direction of movement and speed are likely to remain constant throughout the motion. Furthermore, the randomness in nearly constant model permits the hypothesized targets to change their velocities. This allows us to track targets when there are changes in the velocities of the mobile platform and/or workers.

In RFS approaches, the birth process in a tracking algorithm should be designed in such a way that it captures the newly entering targets as well as those missed. Thus, our birth process is composed of five labeled Bernoulli components, which cover the entire image. Four labeled Bernoulli components are initiated in four sides of the image to detect the newly born targets, since most of the newly born targets appear in the four sides of an image. Another labeled Bernoulli component is initiated in the middle of the image in order to detect the missed targets. This component will enable the detection of targets after occlusion or after reappearing from behind non-target objects (such as walls).

All the birth labeled Bernoulli components have a constant probability of existence of 0.02, and are uniformly distributed within an image. Using additional information, if available, such as positions of the gate entrances, elevator access points, etc., we can incorporate other complex birth models with different probability densities, at the expense of higher computational load. To strike the right balance between accuracy of particle approximation and computation, the number of particles per target are constrained between $L_{\min}=100$ and $L_{\max}=500$.

In the KLA based LMB fusion, the weight ω is set to 0.3. That is, we rely more on the color than the shape features. This enables us to track the targets in partial occlusion—see the results presented in Table 1, Table 2 and Table 3.

At the RMIT University manufacturing workshops, we created a dataset of three video sequences with 346, 835 and 451 frames, respectively. This environment is specifically chosen because it closely resembles an indoor industrial environment; it is rich in visual features such as edges and corners and includes large areas of yellow color close to the color of safety vests worn by industry workers. Each sequence was recorded with a camera attached to a mobile platform which was moved at varying speeds. There are four human targets in each of the sequences and three of them are wearing the safety vest. One person wearing a vest moves randomly, specifically across the camera field of view. The other two wearing safety vests move very close to each other as a group. In two of the sequences, these two persons split after some time and, in the other, they stay together until the end. The person who is not wearing the safety vest also moves randomly. These scenarios are designed to depict the real world scenarios and to test the ability of the proposed trackers to track targets with varying speeds, varying camera ego motion, partial and full occlusions, size variations and different motions within the same sequence.

The main reason for evaluating the performance of the proposed method on our own dataset is that there are no publicly available datasets which contain the human targets who are wearing a high visible vest.

We use various standard tracking and detection metrics to quantify the performance of our proposed methods. We use the set of metrics proposed by Li et al. [46], which have been widely used in the visual tracking literature [47,48,49,50,51]. The metrics include:

– recall (REC - ↑): correctly tracked objects over total ground truth;
– precision (PRE - ↑): correctly tracked objects over total tracking results;
– false alarms per frame (FAF - ↓);
– percentage of objects tracked for more than 80% of their life time (MT - ↑);
– percentage of objects tracked for less than 20% of their life time (ML - ↓);
– percentage of partially tracked objects (PT ↓ = 1 - MT - ML);
– identity switches (IDS - ↓); and
– the number of fragmentations (Frag - ↓) of ground truth trajectories.

Here, the arrow symbol ↑ represents that higher scores indicate better results, and ↓ represents the reverse.

To evaluate the detection performance of our trackers, we use two widely used measures, false negative rate (FNR) and false alarm (positive) rate (FAR). These measures have widely been used in the visual tracking literature [14,15]. They are defined as the following rates: the total number of targets that are missed, and the total number of non-existing but detected targets, divided by the total number of true targets, over all frames. Furthermore, to quantify the tracking performance of our method, following Hoseinnezhad et al. [14,15], we use another two measures, label switching rate (LSR) and lost tracks ratio (LTR). The label switching rate is defined as the number of label switching events occurred during the tracking period normalized by the number of total ground truth tracks. The label switching between two targets can take place when they are moving close to each other and/or after they are separated. If the tracker can not distinguish between close targets, they may be tracked as a single target and hence will have a single label. The lost tracks ratio is defined as the number of tracks which are not detected (misdetected) for more than 50% of their lifetimes, normalized over the total number of ground truth tracks.

In terms of the above noted error measures, we compare our results with the results of the method based on Dynamic Programming and Non-Maxima Suppression (DPNMS method). This is a well-cited state-of-the-art visual tracking technique [52]. The DPNMS method treats multi target tracking as an optimization problem which is solved using greedy algorithms in which dynamic programming and non-maxima suppression is used to omit tracklets representing the same target. Henceforward, the sequential two-step update method is called TSU, and the KLA based LMB fusion method is dubbed KLAF.

To examine the performance of DPNMS method on our datasets, we used the MATLAB code published by the authors of [52]. It is important to note that the target states calculated by DPNMS are rectangular bounding boxes and thus, when calculating the accuracy measures, we had to convert our tracking results for compatibility. That is, we calculated the parameters of the bounding boxes which enclosed the tracked double-ellipse structures. Furthermore, to generate a fair comparison, we manually removed the tracks (generated by DPNMS) that represented the human target not wearing the safety vest.

The results for recall and precision metrics are listed in Table 1. It can be seen that, in terms of the metrics proposed in [47], both of our proposed methods perform better than the state-of-the-art method in comparison. In particular, our Kullback–Leibler method outperforms the other two methods in most of the metric values. It consistently records higher values’ recall and precision metrics, which means that the proposed KLAF method tracks the targets with high accuracy. Furthermore, both of our approaches have not recorded any identity switches for any of the sequences. Most importantly, in a safety critical application such as the application that we focus on in this paper, the KLAF method has not registered any mostly lost targets in any of the sequences. This fact makes the proposed KLAF method suitable for this application.

The detection results and their comparisons are given in Table 2. The table shows that KLAF method outperforms other methods in terms of detection results as well. It should also be noted that our simple approach to sequentially update the predicted multi-target distribution also yields comparative results with respect to the state of the art. In particular, in a safety critical algorithm, the FNR is of high interest and it can be seen that our method has the lowest FNR among the three (which means that it performs better in detecting targets). Except in one particular instance, the KLAF method outperformed the state of the art and the TSU in terms of false alarm rate as well.

The tracking results of our methods and DPNMS methods, in terms of another set of performance metrics, are given in Table 3. In terms of LSR, the proposed KLAF tracker mostly outperformed the other methods (i.e., it distinguished better between targets, even in scenarios where targets were moving very close to each other). Our simple approach, the TSU method is shown to have better performance than the DPNMS method. Furthermore, in one scenario (sequence 02), the DPNMS tracker was not able to track a target for more than 50% of its lifetime while ours have not missed any target for more than 50% of its lifetime. Videos of all the three cases with tracking results are provided as supplemental materials.

**Figure 3:**
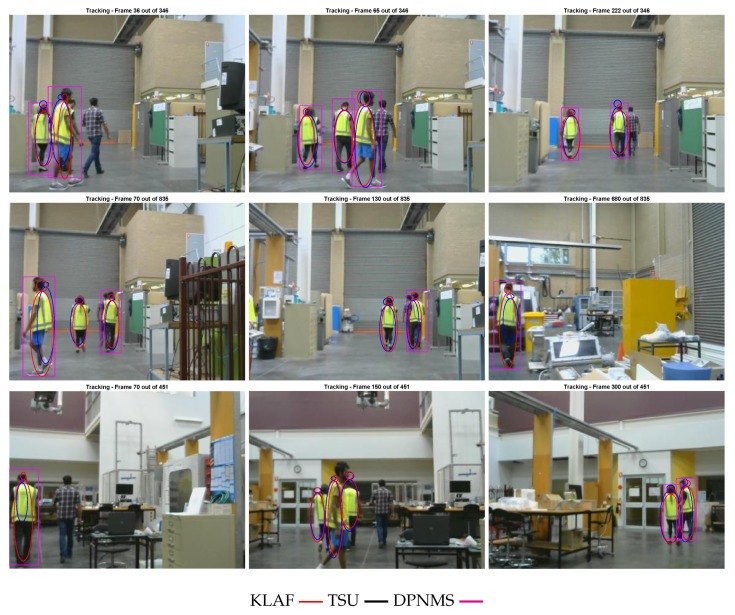
Screen shots of tracking results. Each row is from one sequence. Note: We have omitted the labels of the tracked targets for clarity.

It can be seen in Figure 3 that our trackers perform accurately in cases where the targets are moving close to each other. This demonstrates the efficiency of the merging step of our trackers. Furthermore, all three of the sequences depicted in Figure 3 include a target having a different motion to the other targets, which has been successfully captured by our tracking algorithms.

**Figure 4:**
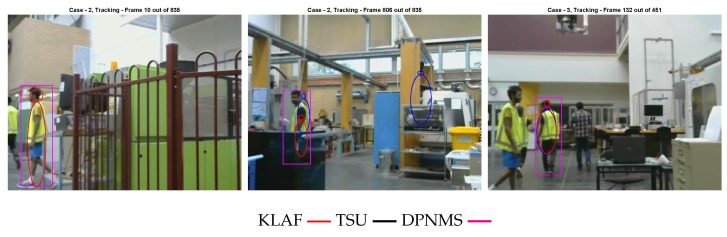
Instances where our methods have failed.

Figure 4 shows a few instances where our methods have failed. The left and right snapshots show two instances where our method has missed a target. This is due to the fact that no birth particle has been generated in those regions of the images, and thus the initialization of those target tracks are delayed. The centre snapshot shows an instance where our sequential update based method has reported a false alarm. This is due to the fact that the particles generated in that region of the image overlap with a yellow colour pole in the background, resulting in higher colour likelihood and edge likelihood (due to the shape of the pole) values for those particles. Thus, their probability of existence is higher and the corresponding hypothesised target track is detected as a valid target at the estimation phase of the filter.

## 6. Conclusions

In this paper, we presented a TBD-LMB algorithm with two approaches to fuse information from different measurements, to track industrial workers who are wearing safety vests. The single target appearance model is designed to capture the geometric shape information of the targets (in the form of a geometric-shape likelihood) along with a kernel density estimation based color likelihood. In one approach, the predicted multi-target distribution is sequentially updated using the specified color likelihood and then by a novel shape likelihood. In the other method, the posterior LMB densities are calculated separately using the aforementioned likelihoods, then fused together using a KLA based fusion method. The comparative results, evaluated on three different video sequences, show that our trackers mostly outperform state-of-the-art in terms of false alarm rate, false negative rate, label switching errors, and losing tracks.

## Figures and Tables

**Table 1:** Comparative results for our dataset with metrics proposed in [47].

Method	Recall%	Precision%	MT%	PT%	ML%	Frag	IDS
KLAF	94.84%	98.49%	100.00%	0.00%	0.00%	1	0
TSU	95.77%	99.45%	100.00%	0.00%	0.00%	1	0
DPNMS	88.76%	99.85%	100.00%	0.00%	0.00%	4	1
KLAF	92.98%	98.68%	100.00%	0.00%	0.00%	2	0
TSU	84.61%	93.39%	67.00%	67.00%	0.00%	2	0
DPNMS	77.38%	83.87%	33.00%	67.00%	0.00%	4	0
KLAF	91.30%	96.78%	100.00%	0.00%	0.00%	3	0
TSU	89.23%	93.22%	100.00%	0.00%	0.00%	2	0
DPNMS	48.48%	87.10%	0.00%	67.00%	33.00%	3	0

**Table 2:** Detection performance for our dataset.

Method	Sequence 01		Sequence 02		Sequence 03	
	FAR%	FNR%	FAR%	FNR%	FAR%	FNR%
DPNMS	0.46	2.45	8.48	9.58	10.86	22.47
TSU	1.25	2.10	9.43	14.39	10.80	12.30
KLAF	0.63	1.54	1.54	4.63	7.13	8.97

**Table 3:** Tracking performance for our dataset.

Method	Sequence 01		Sequence 02		Sequence 03	
	LSR%	LTR%	LSR%	LTR%	LSR%	LTR%
DPNMS	0.86	0.00	0.88	33.33	0.88	0.00
TSU	0.57	0.00	0.44	0.00	1.56	0.00
KLAF	0.46	0.00	0.44	0.00	0.81	0.00
